# ESG performance, herding behavior and stock market returns: evidence from Europe

**DOI:** 10.1007/s12351-023-00745-1

**Published:** 2023-02-15

**Authors:** Nektarios Gavrilakis, Christos Floros

**Affiliations:** Department of Accounting and Finance, Hellenic Mediterranean University, 71410 Heraklion, Greece

**Keywords:** ESG performance, Herding behavior, Market capitalization, Price to book value ratio, Sharpe ratio, Europe

## Abstract

This paper tests how financial performance indicators and combined ESG score for large-cap stocks impact on stock return. In particular, we examine how market capitalization, price to book value, Sharpe ratio and ESG score of large-cap firms in Europe are related to their stock performance. We consider a panel data consisting of six European countries—Portugal, Italy, Greece, Spain, France and Germany—for the period 2010–2020. For Greek and French firms, a firm’s size tends to negatively affect its stock returns. The investors in European countries (except Italy) do not jeopardize their returns by investing in highly ESG scoring firms. We argue that the benefit of not investing in highly ESG-scoring firms might lead investors to select smaller size companies with a higher price to book value and higher Sharpe ratio, as it is more likely to generate higher returns. Moreover, Italian firms are more susceptible to ESG issues, as ESG performance and stock return seem to have a significant negative correlation. This valuable result was confirmed by conducting a robustness test for Europe as a whole using the Euronext100 index. Finally, we find no evidence that ESG motivates herding in our selected sample (this is not the case for Greece and France), while we report evidence of ESG herding behavior during the Covid-19 outbreak in Portugal, Italy and Greece.

**Supplementary Information:**

The online version contains supplementary material available at 10.1007/s12351-023-00745-1.

## Introduction

The role of a sustainable (low-environmental impact) company, in recent years, has become crucial in financial markets, especially in Europe. Therefore, a lot of firms adopt sustainable approaches that combine the evaluation and application of Environmental, Social, and Governance (ESG) factors. The selection and construction of an investment portfolio that is based on sustainable strategies might capture better long-term returns and benefit society by affecting the social conduct of companies. In recent decades, climate changes and social inequality have forced market regulators and policymakers to devote their actions to more sustainable practices based on ESG pillars. Those actions aim to promote the transition to renewable energy and to pivot towards more proactive climate and sustainability-focused positions. According to World Economic Forum (2019), stronger climate action could reduce emissions during the current decade aiming for a balance between emissions and emissions reductions (net-zero transition). The benefits of such climate action could lead to job creation and spur transformational technologies that would be able to help reduce emissions to ‘net zero’ by 2050. At the same time, investors ask companies that focus on sustainability, as represented by the increase of recourses to green bonds and social impact assets (La Torre et al. 2020). PricewaterhouseCoopers (pwc.com) estimates assets under management (AUM) across ESG funds in Europe to be between € 2.6 trillion and € 3.6 trillion by 2025. Deloitte (deloitte.com) reports that by 2025, ESG assets under management will reach close to $35 trillion. As Morningstar Research (Global Sustainable Fund Flows Q2 2022) has noticed, Europe remains the most developed and diverse ESG market with 82% in the 2nd quarter of 2022, followed by the U.S. (12%). As a result, 245 new ESG products entered the global market in line with the 1st quarter of 2022, numbered 242.

The present study is motivated by exploring the current interesting shift of investors and fund managers into ESG strategies by adding to the equation financial factors, like stock performance, firm size (Mcap), firm value (P/BV) and risk value (Sharpe ratio). We chose the above financial factor, as investors and fund managers usually involve them in their investment decisions. Our analysis contributes to the academic debate on financial and ESG issues by extending it to Europe, the biggest market for sustainable funds and addresses a relevant gap in the existing research. The second motive for this study, is to focus on ESG herding in Europe. To our knowledge, this is the first research containing ESG issues and herding behavior in several European countries. ESG herding is the tendency to imitate the observed actions of others, instead of following own beliefs concerning ESG investment decisions. The current increased focus of investors and fund managers on incorporating ESG practices in their asset selection, allocation and diversification of their investment portfolio is called socially responsible investing (SRI) and might capture long-term better returns (Cicireti et al. 2021) or be used as a safe—haven strategy (Rubbaniy et al. 2021) leading companies to follow better corporate governance practices and, in this way, benefit overall the society. Moreover, the increased demand for ESG assets possibly could leads to herd behavior and price anomalies. To study this timely phenomenon is critical not only to herding literature but also to all market participants.

The current study covers two aspects: the impact of financial performance indicators and ESG score on stock return and the influence of ESG score on herding behavior. More precisely, our research is focused on the question of how market capitalization, price to book value, Sharpe ratio and ESG score of large-cap firms in six European countries are related to their stock returns. In addition, the asymmetric behavior of return dispersions concerning ESG performance is evaluated.

A two-step methodology is used to analyze the performance of companies of Portugal (PSI20), Italy (FTSEMIB), Greece (FTSE/Athex20), Spain (IBEX35), France (CAC) and Germany (DAX) indices over the 2010–2020 period according to their ESG score. In addition, Portugal (PSI20), Italy (STOXX Italy45 ESG), Greece (ATHEX ESG), Spain (STOXX Spain30 ESG), France (CAC40 ESG) and Germany (DAX50 ESG) ESG indices were analyzed. ESG data were collected from the Refinitiv Eikon database. Refinitiv ESG scores mirror firm disclosure on environmental, social, and governance metrics (Refinitiv 2020).

In contrast to stock markets, for which research is abundant, the literature about the investor’s trend in ESG stock markets is scant. The present study contributes to this debate by empirically investigating not only the relationship between ESG performance and stock return but also the herding behavior of ESG stocks in European countries. Our empirical investigation provides evidence in support of the mediocre role of ESG disclosure. More specifically, our results show that, concerning European companies, price to book value and Sharpe ratio have a significant positive impact on stock returns. Furthermore, the size of the company is negatively correlated with stock returns for Greece and France. Italy is sensitive about EGS issues by sacrificing stock returns. As a robustness check, we performed the same data model estimations for the Euronext100 index, which consists of the biggest and more liquid stocks traded on Euronext. The findings are broadly in line with the results of the European countries involved in the study. However, it is established that Europe as a whole is more sensitive to ESG investing. Finally, no evidence is found that ESG motivates herding in our selected sample (this is not the case for Greece and France), while evidence of ESG herding behavior during the Covid-19 outbreak in Portugal, Italy and Greece is documented. In periods of market distress, investors are more prone to herding and follow crowd decisions. The unexpected shock of Covid-19 pandemic on the countries’ economic growth shifted into an adverse shock to the stock markets by increasing volatility and trading volume leading to asset mispricing and market inefficiency.

The contribution of our study is crucial to the contemporary literature by advancing investment decision-making of investors and portfolio managers implementing ESG investment strategies. Also, the financial performance of organizations is increasingly affected by financial and environmental factors and thus, the current study has essential implications for companies that focus on sustainability. Moreover, we contribute to the herding behavior literature by emphasizing the association of ESG herding bias with portfolio diversification and performance. Finally, the outcomes of our study have important implications for market regulators to formulate their regulatory ESG policies.

The remainder of this article is structured as follows. In the next section, we review the literature and explain ESG performance. Section 3 describes the methodology and the data. The results are reported in Sect. 4, while Sect. 5 concludes the paper.

## Literature review

Starting from the main objective of the conducted study, four factors have been defined: Market capitalization (Mcap), Price to Book Value (P/BV), Sharpe ratio (Sharpe) and Environmental Social Governance (ESG) factors. Those determinants are very crucial not only for the financial performance of the company but also for investors and fund managers in the process of stock picking and portfolio construction.

Market cap or market capitalization is defined as the total size of a company in the stock market. The knowledge of Mcap is critical to building a portfolio with assets of different size mixes. In their study, Sanches et al. (2016), found that companies in sensitive industries (SI) produce better ESG performance considering BRICS countries, even when the size (Mcap) of the company and the country are controlled. Akgun et al. (2021) reinforces the idea that ESG scores have the potential to increase portfolio performance in US small cap stocks. In their study, Engelhardt et al. (2021), examines the relationship between ESG ratings and stock performance during the COVID-19 crisis and come up with a positive and statistically significant coefficient on market capitalization (size), ROE, market to book ratio, historical volatility and momentum. In addition, they deduce that high ESG-rated European firms relate to lower stock volatility and higher abnormal returns.

The price to book value ratio (also called market to book ratio) is a metric used mostly in value investing to evaluate the present market value of an asset relative to its book value. Market-based measures, such as P/BV mirror the market’s consensus of both contingent and present profitability and capture the indefinable value in the stock market above the book value of the company (Hassel et al. 2013). Book value adjusted for inflation has a stronger relationship with asset values as Anandarajan et al. (2006) point out in their study. Investors and asset managers should give more attention to P/BV than earnings in periods with high inflation. In another study, Ionescu et al. (2019) examined the link between ESG and firm market value (price to book ratio) and identified that the most important influence on P/BV has the governance factor of a global sample of companies from the travel and tourism industry. Moreover, Chiu et al. (2021) study in China listed companies concluded that assets selected by lower P/BV had better performance than traditional investment strategies.

The meaning of diversification is vital when constructing an investment portfolio. Diversification is the process of portfolio construction by investing in different asset classes with the main objective to minimize the overall risk. Sharpe ratio (Sharpe 1963) is a method of spreading the risk without inducing the return negative and constitutes one of the most used risk and return metrics in portfolio management. Sharpe ratio can elucidate if the return of an asset is a reason for “smart investment decisions” or if the investor has taken a high risk (Bodie et al. 2011). In other words, Sharpe ratio is the average return earned over the risk-free rate after adjusting for its risk. Kumar, et al. (2016) analyze if ESG score impacts the volatility of U.S. stocks. They found Sharpe ratio for ESG companies is greater than their compeers in the same sector and reported that firms with higher ESG scores create higher returns. Moreover, they deduced that investors could have earned a higher-than-average return for each unit of risk taken if they had invested in stocks with a high ESG score. Fatemi et al. (2018) argued that the companies with a lower level of risk and good ESG practices are inclined to have more dedicated employees, a lower probability of lawsuits, more loyal customers, and as a result higher company valuation level. Caner et al. (2022) analyzed the consistency of Sharpe ratio estimator and concluded that the Sharpe risk indicator is consequent in minimum and mean–variance portfolios.

In the past several years, ESG has growing attention in the academic, business and political realms. ESG is the use of Environmental Social and Governance (ESG) non-financial factors in evaluating the progress of companies on sustainability practices. A lot of studies have attempted to estimate the impact of ESG performance of a firm on stock return. Results from research, conducted in ESG disclosure, are conflicting with some of them indicating that there is a positive impact of ESG on financial performance. Others find the impact to be negative, while recent papers find no impact at all. The contradicting findings provide a research gap that the current article examines using data from Europe. A better understanding of ESG importance in our changing world will benefit investors, companies and the society overall.

In their study, Clark et al. (2015) concluded that firms that develop sustainable strategies yield greater financial returns. In addition, investors taking sustainability into account can deliver improved investment performance. Nagy et al. (2016) argue that MSCI's ESG stocks portfolio outperformed the MSCI World index over the sample period of 2007–2015. Moreover, El Ghoul and Karoui (2017) conclude that ESG performance and firm value are positively related, especially in countries with weaker market-supporting organizations. Velte (2017) presents evidence that ESG has a positive effect on return on assets (ROA), but no effect on firm value (measured by Tobin’s Q). ESG increases the financial performance of German companies in short term, but not in the long run, as Velte (2017) identified. In their study, Dalal and Thaker (2019) find that good ESG practices increase financial performance measured with ROA indicator and Tobins’s Q factor, on a panel data of Indian companies. Their results indicate that investors prefer socially responsible companies. The results of Alsayegh et al. (2020) study show that there is a positive relationship between environmental and social performance among Asian companies for the period between 2005 and 2017. Moreover, Borokova and Wu (2020) examine the performance of U.S. companies during the Covid-19 outbreak and report that companies with low ESG scores noted 50% lower returns than those with high ESG scores. Recently, Broadstock et al. (2021) investigated a sample of Chinese firms to conclude that portfolios with more ESG assets outperform portfolios with fewer ESG assets during Covid-19 pandemic.

Based on the related literature, some studies indicate a negative relationship between ESG score and stock return. Fisher-Vanden and Thorburn (2011) found that companies announcing membership in the Environmental Protection Agency (EPA), a center for corporate climate leadership, experience significant negative abnormal stock returns. Sahut and Pasquini-Descomps (2015) provide evidence of a negative relationship between ESG and financial performance using news-based ESG scores in the U.S., U.K. and Switzerland. In another study, Fatemi et al. (2015) examines the impact of environmental social and governance actions on firm value and found that strong ESG practices elevate firm value and ESG concerns diminish it. In addition, when ESG is isolated, then the firm value is decreased.

Landi and Sciarelli (2019) examine if ESG rating influences the stock returns of Italian companies and conclude in a non-positive significant effect. Moreover, a negative or low correlation of ESG securities with other assets; stocks, currency and commodities, during normal and crisis periods allows investors to hedge their portfolios using ESG stocks (Ferriani and Natoli 2020; Andersson et al. 2020). By considering 85 different studies for 20 years, Revelli and Viviani (2015) deduce that there is no significant relationship between stock performance and ESG. Halbritter and Dorfleitner (2015) examined the correlation between ESG ratings and financial performance by using ESG data for the U.S. market from 1991 to 2012 and found that portfolios with high and low ESG rating companies have no significant return differences. Another study conducted by Limkriangkrai et al. (2017) on large-cap stocks in Australia showed that portfolios based on ESG ratings have no significant difference in risk-adjusted returns. There is no relationship between climate change policies and emission reductions with the financial performance of large-cap U.S. companies as identified by Petitjean (2019). Moreover, La Torre et al. (2020) investigate how ESG score affects the stock performance of companies that constitute the Eurostoxx50 index, over the 2010–2018 period and concluded that the performance does not seem to be influenced by the company’s actions in terms of ESG commitments. Based on the review of the literature, the current article examines the following hypothesis for six European countries.

### H1

Market capitalization, Price to book value, Sharpe ratio and ESG performance affect stock returns.

The review of the literature leads to the second contribution of this study, the investigation of herding behavior on ESG stocks. The current advanced demand for ESG assets might lead investors and fund managers to herd behavior (Przychodzen et al. 2016; Benz et al. 2020; Rubbaniy et al. 2021). Most of the time inexperienced investors follow the investment behavior of others who are believed to be “market experts”. At the same time, the growing significance of ESG investment practices drives many investors to take “personal action” by adding constantly ESG assets to their portfolios without taking care of the diversification. This non-rational behavior creates herding that can cause massive inflows into particular asset classes like ESG by creating a market bubble. In addition, lower or non-exposure to ESG investing during days of market stress (D’Hondt et al. 2022) might mean that investors don’t prefer ESG assets on crisis periods. This behavior could create ESG herding leading to a market crash. In her study, Blondel (2022) found that medium-risk profile investors herd for traditional investments more than ESG investments. Regarding ESG investments, passive investors seem to herd more than active investors as Blondel (2022) concluded. Finally, in their study, Youssef et al. (2022) examined the impact of COVID-19 crisis on herding behavior in the cryptocurrency market and noticed the existence of herding during the COVID-19 crisis.

Scharfstein et al. (1990) evidenced that when the performance of fund managers is assessed relative to others, the fund managers who lack confidence are apprehensive of underperforming and thus are more intense to mimic the trading behavior of other fund managers, who are more experienced and qualified. Walter et al. (2006) detects spurious herding (when investment decisions are alike but based on an independent analysis by professionals) of mutual fund managers in Germany as an aftereffect of changes in benchmark index composition. Moreover, Choi et al. (2009) document strong institutional herding in U.S. companies. Przychodzen et al. (2016) investigate the behavior, motives and characteristics of mutual fund managers who incorporate ESG strategies into their investment decisions by providing evidence of herding behavior. In their study, Benz et al. (2020) analyze institutional ownership data and conclude that investors, portfolio advisors and hedge funds follow herding behavior when decarbonizing their portfolios. Furthermore, they concluded that Institutional investors (mutual funds and hedge funds) follow ESG herding due to anxiety to accomplish the market return consensus by imitating their peer’s investment strategies. Similarly, Pension funds and Insurance companies are committed to societal norms and values driving them to invest constantly in ESG assets. Moreover, by using data of the MSCI U.S.A. ESG leader index, Rubbaniy et al. (2021) identify herding behavior during both bear and bull market conditions over the period 2007–2020.

On the other hand, Babalos and Stavroyiannis (2015) confirm an anti-herding behavior in metal commodities futures before the global financial crisis. Rompotis (2018) examined a sample of 66 large-cap and 34 small-cap exchange-traded funds (ETFs) and found no herding effect over the period 2012–2016. Demirer et al. (2019) concluded that small firms with a high level of herding underperform those small firms that experience a low level of herding. More recently, Yarovaya et al. (2021) find no evidence of herding in cryptocurrencies during Covid-19, but report that herding is contingent upon the up and down-market conditions. Finally, using a dataset of 10, 456 unique global ESG funds from 2012 to 2018, Ciciretti et al. (2021) found that ESG funds exhibit an anti-herding behavior. Based on the previous review, some studies show a positive relationship between herding and ESG performance, while others show no herding effect. By focusing on ESG herding our study contributes to the academic discussion on herd behavior by examining this phenomenon in Europe. The above findings lead us to formulate the following hypothesis for six European countries:

### H2

Companies adopting ESG practices do not necessarily induce herding behavior.

## Methodology and data description

**Fig. 1:**
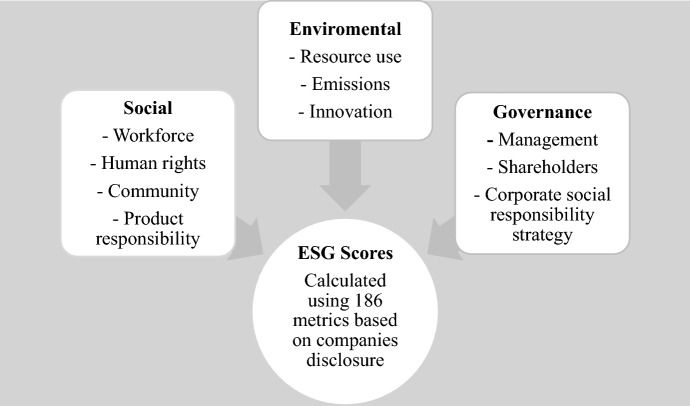
Overview of categories and subcategories in Refinitiv ESG data (Borokova and Wu 2020)

We use Refinitiv’s database for ESG scores as it takes into consideration combined scores (Refinitiv 2020). DataStream Refinitiv Eikon is chosen for retrieving yearly data since it provides one of the most inclusive databases, covering over 80% of the global market cap across more than 450 different ESG measures (Breitz and Partapuoli, 2020). These measures are divided into three main categories and ten subcategories. The categories are Environment (E), Social (S) and Governance (G). ESG scores were calculated using a subset of 186 metrics. The overview of ESG score is shown in Fig. 1.

Following Borokova and Wu (2020) study, we do not perform our analysis separately for E, S and G scores but we use the overall Refinitiv combined ESG scores as Borokova and Wu (2020) implement in their study.

In the first part, we follow Borokova and Wu (2020) regression model with changes not only in the financial indicators but also in the selected sample of countries. We place ESG performance in a framework, along with financial indicators such as Market capitalization (Mcap), price to book value (P/BV) and Sharpe ratio (risk measure) to identify any relationships with stock return. Table 1 presents the estimation formulas for the dependent and independent variables used in the analysis.

**Table 1:** Methodology used for the construction of dependent and control variables

Dependent variable	Measurement
Rit (Stock return)	The total return is the amount of value an investor earns from a security over a specific period. It is expressed as a percentage of the amount invested.
*Independent variables* Mcap (Size)	Market cap (market capitalization) is the total value of all a company's shares of stock. It is calculated by multiplying the price of a stock by its total number of outstanding shares.
P/BV (Valuation)	Price to Book Value is calculated by dividing the company’s latest closing Price by its Book Value per share.
Sharpe ratio (Risk)	The Sharpe ratio is the average return earned over the risk-free rate after adjusting for its risk.
ESGt (Environmental Social Governance) score	ESG Combined Score is an overall company score based on the reported information in the environmental, social and corporate governance pillars (ESG Score) (Refinitiv 2020).

All data were retrieved from Refinitiv Eikon Database

According to Borokova and Wu (2020), financial literature on ESG and financial performance define two dimensions: those who believe that ESG actions sacrifice financial returns and those who believe that ESG practices ensure lower risk and better returns in the long run. Regarding the relationship between ESG performance and stock returns most of the carried-out studies are limited to U.S. companies. The current study determines the above relationships in European companies**.** The stocks used is the set of constituents of the PSI20, FTSEMIB, FTSE/Athex20, IBEX35, CAC and DAX indices. As a robustness check, we performed the same data model estimations for the Euronext100 index. The total period analyzed was from 04/01/2010 to 31/12/2020.

The second part of this study examines if ESG performance leads to herding behavior across Europe. We apply the cross-sectional dispersion approach to examine the herding behavior using yearly observations on closing prices and ESG scores of stocks that constituent PSI20, FTSEMIB, FTSE/Athex20, IBEX35, CAC, DAX large-cap indices. This study investigates herding behavior by focusing on ESG performance. The sample period stretches from 04/01/2010 to 31/12/2020. We take more than 10 years of data to examine herding behavior and ESG performance, as investors prefer to have enough time to analyze markets and gather financial information to make rational investment decisions according to Mertzanis and Allam (2018). Furthermore, we surveyed Portugal (PSI20), Italy (STOXX Italy45 ESG), Greece (ATHEX ESG), Spain (STOXX Spain30 ESG), France (CAC40 ESG) and Germany (DAX50 ESG) ESG indices to analyze herding behavior during the Covid-19 period. All tested constituents of all indexes encompass over 285 large-cap firms. Company financial indicators are retrieved from DataStream Refinitiv Eikon.

### Methodology of stock return and ESG performance

Multiple regression analysis was used to unfold the variables that have the most and least influence on the stock returns of the selected indices. Our dependent variable is the stock return *Rit* of the stock *i* in year *t.* The panel data regression model is:

1 $${R}_{it}=a+{\beta }_{1}\left(Mcap\right)+{\beta }_{2}\left(P/BV\right)+{\beta }_{3}\left(Sharpe\right)+{\beta }_{4}\left({ESG}_{t-1}\right)+{\varepsilon }_{it}$$

where, *Mcap* is the market capitalization of asset, *P/BV* is the price to book value ratio*, Sharpe* is the Sharpe ratio and *ESGt-1* is the yearly ESG performance. The determinants are financial variables (for size, value and risk) that investors and professionals mostly analyze before their investment decision. We use a combined ESG score, as this is the typical score’s update more frequently. The terms *α* (constant) and *β* (regression coefficient) are parameters to be assessed and ${\varepsilon }_{it}$ is the error term.

### Methodology of herding behavior and ESG performance

For herding behavior, we employ the most common measure of return dispersion in the herding literature, originally, proposed by Chang et al. (2000) and related to the cross-sectional absolute deviation of returns (CSAD). A linear relation between dispersion and market returns does not hold in times of large price movements according to Chang et al. (2000). Therefore, herd behavior during periods of large price movements converts the linear relation into a non-linear one. In other words, the cross-sectional absolute deviation will decline or at least increase at a less-than-proportional rate with the market return. To capture this effect, we estimate the cross-sectional absolute deviation (CSAD) formula (2) as an estimator of return dispersion as follows:

2 $$CSAD_{t} = \frac{1}{N}\sum\limits_{i = 1}^{N} {\left| {R_{i,t} - R_{m,t} } \right|}$$

where *R*_*i,t*_ is the return of the *ith* stock on day *t*, *R*_*m,t*_ is the market return on the same day and *N* is the number of stocks in the index used. A lower or a less than proportional increase in CSAD should be expected if stocks herd during market stress (Rompotis 2018).

By Chang et al. (2000), the non-linear relationship between stock return dispersion and market performance is estimated following formula (3):

3 $$CSAD_{t} = \alpha + \gamma_{1} \left| {R_{m,t} } \right| + \gamma_{2} R_{m,t}^{2} + \varepsilon_{t}$$

where *R*_*m,t*_ is defined as above while *R*^*2*^*m,t* is used to capture the non-linearity in the relationship. In the absenteeism of any herding effects, *γ*_*1*_ coefficient will be positive and *γ*_*2*_ coefficient will be equal to zero. According to Rompotis (2018), a significantly negative coefficient *γ*_*2*_ will display the phenomenon of herding behavior, since it mirrors the fact that during market distress, a nonlinear negative relationship between return dispersion and *R*^*2*^*m,t* exists. To detect whether there is any non-linearity between the CSAD of Chang et al. (2000) return dispersion measure with the market, we apply model (3) having CSAD as the dependent variable of the model.

Moreover, the asymmetric behavior of return dispersions concerning ESG performance can be estimated as follows:

4 $$CSAD_{t} = \alpha + \gamma_{1} \left| {R_{m,t} } \right| + \gamma_{2} R_{m,t}^{2} + \gamma_{3} ESG_{{}} (R_{m,t}^{2} ) + \varepsilon_{t}$$

where *R*_*m,t*_ is defined as above and *ESG* is the main average performance of ESG score. ESG performance is expressed as decimal rather than a percent (so it is divided by 100), to share the same measurement as other independent variables. In the absenteeism of any herding effects, *γ*_*1*_ coefficient will be positive and *γ*_*2*_ coefficient will be equal to zero. As Guney et al. (2017) pointed out, significant negative values of *γ*_*2*_ indicate the existence of ‘‘domestic” herding, in market *m*, while a significant negative coefficient *γ*_*3*_ indicates that herding in market *m* is stimulated by the presence of ESG score. Newey and West (1987) heteroscedasticity and autocorrelation consistent estimators were used to addressing the autocorrelation issue in the estimates of regression coefficients.

Finally, by Christie and Huang (1995), herding is more likely to appear during periods of extreme market movements, meaning that herding is more dominant during market crises. Our study examines whether herding effects are more pronounced during the period of the Covid-19 pandemic. The asymmetric behavior of return dispersion concerning Covid-19 is assessed as follows:

5 $$CSAD_{t} = \alpha + \gamma_{1} \left| {R_{m,t} } \right| + \gamma_{2} R_{m,t}^{2} + \gamma_{3} D^{cov} (R_{m,t}^{2} ) + \varepsilon_{t}$$

where *R*_*m,t*_ is defined as above and *D*^*cov*^ is a dummy variable taking value 1 for Covid-19 period and 0 otherwise. We chose Covid-19 outbreak period from 30th January 2020 to 1st June 2020, as the volatility of STOXX ® Europe 600 ESG—X index (one of Europe's ESG key benchmarks) reached levels before the 30th of January when the World Health Organization (WHO) announced the Covid-19 pandemic as a public health emergency of international concern.

In the absence of any herding effects, *γ*_*1*_ coefficient will be positive and *γ*_*2*_ coefficient will be equal to zero. According to Guney et al. (2017), significant negative values of *γ*_*2*_ would indicate the existence of significant ‘‘domestic” herding, in market *m,* while a significant and negative value for coefficient *γ*_*3*_ will be consistent with herding behavior during Covid-19 outbreak.

## Empirical results

Table 2 presents the estimated results of model (1) for stocks in Portugal (PSI20), Italy (FTSEMIB), Greece (FTSE/Athex20), Spain (IBEX35), France (CAC) and Germany (DAX). The results show some important statistics concerning the independent variables and their eligibility in influencing the dependent variable. Accordingly, the coefficient of Market capitalization (*Mcap)* for Greece and France is respectively − 4048 and − 5.742, which means that a 1 unit change in *Mcap* impacts − 4048 and − 5.742 units change in the stocks’ return, respectively. This significant and negative relationship between Market capitalization and stock return, possibly suggests that investors prefer smaller companies that may generate higher returns. Furthermore, in all countries of the panel (except Portugal) price to book value (*P/BV*) and Sharpe ratio elevate the returns, as the comparable coefficients are positive and significant. All countries except Italy do not offer up their stock returns, as we do not detect a significant relationship between ESG scores and firm returns. This is a result of less pivot on sustainability for the selected countries (except Italy), probably leading to a dropdown in demand by investors for stocks with high ESG scores. Our results contradict Sahut and Pasquini-Descomps (2015), who found a negative relationship between ESG and financial performance in U.S., U.K. and Switzerland. Regarding Italy, our results are in line with Landi and Sciarelli (2019), who observed a negative impact of excess return and socially responsible investment. In addition, we partly, confirm the results suggested by Borokova and Wu (2020), who identified no significant relationship between ESG scores and returns in Australian and South Korean firms. Finally, our findings are contrary to the results from the study of Borokova and Wu (2020), who found a significant negative relationship between ESG scores and excess returns in U.S. and Asian companies.

**Table 2:** Regression results for Portugal (PSI20), Italy (FTSEMIB), Greece (FTSE/Athex20), Spain (IBEX35), France (CAC) and Germany (DAX)

A. Portugal (PSI20) * M.A. ESG score: 62.17 (B)					B. Italy (FTSEMIB) M.A. ESG score: 57.69 (B-)				
	Coeff	SD	T-ratio	P		Coeff	SD	T-ratio	P
Mcap	− 3.273	6.943	− 0.615	0.522	Mcap	1.607	1.212	1.326	0.193
P/BV	0.021	0.017	1.219	0.250	P/BV	0.044	0.010	4.386	0.001***
Sharpe	0.227	0.190	1.195	0.259	Sharpe	0.583	0.111	5.243	0.3e-6***
ESG	− 0.257	0.141	− 1.813	0.099	ESG	− 0.147	0.080	− 1.832	0.057*
* Small sample size									
C. Greece (FTSE/Athex20) M.A. ESG score: 57.69 (B-)					D. Spain (IBEX35) M.A. ESG score: 65.34 (B)				
	Coeff	SD	T-ratio	P		Coeff	SD	T-ratio	P
Mcap	− 4.048	0.000	− 2.150	0.046**	Mcap	− 5.063	5.872	− 0.862	0.397
P/BV	0.090	0.027	3.296	0.004***	P/BV	0.034	0.010	3.362	0.002***
Sharpe	0.727	0.173	3.201	0.001***	Sharpe	0.413	0.189	2.181	0.039**
ESG	− 0.070	0.177	− 0.393	0.699	ESG	− 0.058	0.171	− 0.339	0.737
E. France (CAC) M.A ESG score: 64.80 (B)					F. Germany (DAX) M.A. ESG score: 64.79 (B-)				
	Coeff	SD	T-ratio	P		Coeff	SD	T-ratio	P
Mcap	− 5.742	2.813	− 2.039	0.0493**	Mcap	− 5.063	5.872	− 0.862	0.397
P/BV	0.022	0.004	4.585	5.89e-05***	P/BV	0.034	0.010	3.362	0.002***
Sharpe	0.304	0.048	6.347	3.07e-07***	Sharpe	0.413	0.189	2.181	0.039**
ESG	− 0.038	0.083	− 0.468	0.642	ESG	− 0.058	0.171	− 0.339	0.737

Table 1 reports the estimated coefficients for the following model: ${R}_{it}=a+{\beta }_{1}\left(Mcap\right)+{\beta }_{2}\left(P/BV\right)+{\beta }_{3}\left(Sharpe\right)+{\beta }_{4}{ESG}_{t-1}+{\varepsilon }_{it}$. *Rit* is the stock return*, Mcap* is market capitalization, *P/BV* is price to book value ratio*, Sharpe* is the Sharpe ratio and *ESG* is the yearly ESG performance. *** Significant at 1%, ** Significant at 5% and * Significant at 10%

We performed the same data model estimations, as a robustness check, for Euronext100. The Euronext100 Index is the blue-chip index of the Euronext NV (pan-European exchange). Table 3 presents the estimation results of model (1) for stocks in Euronext100. The regression equation for European companies becomes:

$${\varvec{R}}_{{{\varvec{it}}}} {\mathbf{ = 0,124 - 5,342}}\left( {{\varvec{Mcap}}} \right){\mathbf{ + 0,016}}\left( {{\varvec{P}}{\mathbf{/}}{\varvec{BV}}} \right){\mathbf{ + 0,335}}\left( {{\varvec{Sharpe}}} \right){\mathbf{ - 0}}{\mathbf{.095}}\left( {{\varvec{ESG}}_{{{\varvec{t}}{\mathbf{ - 1}}}} } \right) {\mathbf{ + }}{\varvec{\varepsilon}}_{{{\varvec{it}}}}$$

**Table 3:** Regression results for Euronext100

$${\mathrm{Model summary}}$$					
Model	R	R^2^	Adjusted R^2^	Std. error	
	0.727	0.529	**0.508**	0.066	

$${\mathrm{ANOVA}}$$					
Model	Sum of Sq	df	Mean Sq	F	Sig
Regression	0.448	4	0.112	25,253	**0.000**
Residual	0.399	90	0.004		
Total	0.846	94			

$${\mathrm{Coefficients}}$$					
Model	Unstandardized coefficients		Beta	t	Sig
	B	Std. error			
Constant	0.124	0.033		3.767	0.000
**Mcap**	− 5.342	0.000	− 0.189	− 2.605	0.011**
**P/BV**	0.016	0.004	0.330	4.450	0.000***
**Sharpe**	0.335	0.048	0.522	7.045	0.000***
**ESGt-1**	− 0.095	0.049	− 0.140	− 1.923	0.0576*
*Dependent Variable: Ri/Predictors: Mcap, P/BV, Sharpe ratio, ESGt-1/N* = *100*					

Table 2 reports the estimated coefficients for the following model: ${R}_{it}=a+{\beta }_{1}\left(Mcap\right)+{\beta }_{2}\left(P/BV\right)+{\beta }_{3}\left(Sharpe\right)+{\beta }_{4}{(ESG}_{t-1})+{\varepsilon }_{it}$. *Rit* is the stock return*, Mcap* is market capitalization, *P/BV* is the price to book value ratio*, Sharpe* is the Sharpe ratio and *ESG* is the yearly ESG performance. *** Significant at 1%, ** Significant at 5% and * Significant at 10%

Taking all the above variables of the regression equation constant at zero, the performance of the stock would be positive (0,124). According to the results, the coefficient of Market capitalization (*Mcap*) is − 5342, which means that a 1 unit change in *Mcap* impacts − 5342 units change in the stocks’ return. This significant and negative relationship between the Market capitalization and stock return, suggests that European investors possibly select smaller companies in their portfolios as it is more likely to yield higher returns on average. Furthermore, European stocks with a higher price to book value (*P/BV*) and Sharpe ratio, increase the returns, as the relative regression coefficients are positive and significant. Finally, European stocks immolate their stock returns as for those companies we identify a marginally significant negative relationship between ESG scores and returns, even though, there is an increasing demand by investors for companies with high ESG scores as Sunniva (2021) has noticed in his research. This finding is consequent with the outcome of the study of Sahut and Pasquini-Descomps (2015) who found evidence of a negative relationship between ESG and financial performance. Moreover, our result partly confirms Borokova and Wu (2020) who found that Asian firms are more alike to U.S. ones as firms with high ESG scores tend to have next year lower returns. Contrary, our findings do not support the study of La Torre et al. (2020) who identified that Eurostoxx50 companies’ performance does not seem to be influenced by their actions concerning ESG responsibility.

The results for Euronext100 are broadly in line with the results rendered by the panel of the countries used in the study. Moreover, the robustness test for Europe as a whole (Euronext100 index) shows that ESG performance and stock returns seem to have a significant negative correlation. The above result implies that a 1 unit change in ESG score impacts − 0095 unit change in stock’s return. This significant and negative relationship between ESG performance and stock return suggests that European investors possibly sacrifice stock returns for companies with good ESG performance.

Regarding the second part of the current study, Table 4 presents the estimation results of the model (4) by applying the cross-sectional absolute deviation of returns (CSAD) by Chang et al. (2000). We examine the herding behavior using yearly observations on closing prices and ESG scores of stocks that constituent PSI20, FTSEMIB, FTSE/Athex20, IBEX35, CAC and DAX indices. The results indicate that ESG performance motivates herding in Greek and French companies, as there is a significantly negative coefficient *γ3*. A significantly negative coefficient *γ3* indicates that herding in the Greek and French market is motivated by the presence of ESG scores. We confirm Benz et al. (2020) who found herding behavior in investors, portfolio advisors and hedge funds when decarbonizing their portfolios. Furthermore, we partly support the results of Blondel (2022) who indicates that passive investors herd more than active investors. The outcomes for the rest countries indicate that ESG performance does not motivate herding, as there is no significant negative coefficient *γ3*. It seems that ESG investors do not imitate and observe the trading actions of others (herding behavior) but rely on their own beliefs and information mostly because ESG strategies are for long-term investments.

**Table 4:** Herding analysis for Portugal, Italy, Greece, Spain, France, Germany

$$CSAD_{t} = \alpha + \gamma_{1} \left| {R_{m,t} } \right| + \gamma_{2} R_{m,t}^{2} + \gamma_{3} ESG_{{}} (R_{m,t}^{2} ) + \varepsilon_{t}$$				
Model	Unstandardized coefficients		t	Sig
	B	Std. error		
*A. Portugal (PSI20)*				
*Constant*	0.014	0.001	13.31	1.11e-05***
*|Rm,t|*	− 0.041	0.016	− 2.578	0.014**
*R*^*2*^*m,t*	− 0.570	0.626	− 0.916	0.397
*ESG(R*^*2*^*m,t)*	1.144	1.052	1.088	0.318
*B. Italy (FTSEMIB)*				
*Constant*	0.009	0.005	1.849	0.113
*|Rm,t|*	0.251	0.111	2.264	0.064*
*R*^*2*^*m,t*	− 1.065	0.972	− 1.095	0.315
*ESG(R*^*2*^*m,t)*	0.330	1.196	0.276	0.791
*C. Greece (FTSE/Athex20)*				
*Constant*	0.015	0.004	3.398	0.014**
*|Rm,t|*	0.074	0.043	1.735	0.133
*R*^*2*^*m,t*	0.756	0.232	3.256	0.0173**
*ESG(R*^*2*^*m,t)*	** − 1.702**	0.512	− 3.324	0.0159**
*D. Spain (IBEX35)*				
*Constant*	0.019	0.002	7.703	0.000***
*|Rm,t|*	− 0.169	0.057	− 2.922	0.026**
*R*^*2*^*m,t*	1.187	0.872	1.361	0.222
*ESG(R*^*2*^*m,t)*	− 0.464	1.617	− 0.287	0.783
*E. France (CAC)*				
*Constant*	0.009	0.000	15.03	5.48e-06***
*|Rm,t|*	− 0.047	0.021	− 2.263	0.064*
*R*^*2*^*m,t*	1.149	0.395	2.903	0.027**
*ESG(R*^*2*^*m,t)*	** − 1.416**	0.479	− 2.951	0.0256**
*F. Germany (DAX)*				
*Constant*	0.009	0.002	3.097	0.006***
*|Rm,t|*	− 0.005	0.033	− 0.167	0.872
*R*^*2*^*m,t*	0.028	0.055	0.503	0.6323
*ESG(R*^*2*^*m,t)*	− 0.030	0.136	− 0.223	0.830

Table 3 reports the estimated coefficients for the following model: $CSAD_{t} = \alpha + \gamma_{1} \left| {R_{m,t} } \right| + \gamma_{2} R_{m,t}^{2} + \gamma_{3} ESG_{{}} (R_{m,t}^{2} ) + \varepsilon_{t}$. *CSAD*_*t*_.is the cross-sectional absolute deviation of stock returns concerning cross sectional average return *Rm,t. ESG* is the main average performance of ESG score. *** Significant at 1%, ** Significant at 5% and * Significant at 10%. All estimations use the Newey and West (1987) heteroscedastic and autocorrelation corrected standards errors.

Our results are in line with Rompotis (2018) who analyze a sample of ETFs. He reports no evidence of herding. In addition, our results are in line with Yarovaya et al. (2021) who find no evidence of herding in cryptocurrencies during Covid-19; they also report that herding is contingent upon the up and down-market conditions. Finally, our results are not consistent with Benz et al. (2020) who analyze the decarburization of institutional portfolios and show that investors follow herding behavior when they decarbonize their portfolios.

The recent Covid-19 outbreak is believed to affect global financial markets. Table 5 presents the estimation results of the model (5) by applying the cross-sectional absolute deviation of returns (CSAD) as suggested by Chang et al. (2000). We examine the herding behavior using daily observations on closing prices of ESG stocks that constituent PSI20 (we chose highly ESG scoring companies in the luck of ESG index), STOXX Italy45 ESG, ATHEX ESG, STOXX Spain30 ESG, CAC40 ESG and DAX50 ESG indices. The total period analyzed was from 04/01/2010 to 31/12/2020.

**Table 5:** Herding analysis for European ESG indices during Covid-19

$$CSAD_{t} = \alpha + \gamma_{1} \left| {R_{m,t} } \right| + \gamma_{2} R_{m,t}^{2} + \gamma_{3} D^{{\text{cov}}} (R_{m,t}^{2} ) + \varepsilon_{t}$$				
Model	Unstandardized coefficients		t-ratio	Sig
	B	Std. error		
A. Portugal (PSI20)_*High scored ESG stocks				
*Constant*	0.008	0.000	69.85	0.000***
*|Rm,t|*	0.272	0.019	14.30	0.000***
*R*^*2*^*m,t*	0.506	0.536	0.944	0.345
*D*^*cov*^* (R*^*2*^*m,.t)*	** − 1.186**	0.583	− 2.035	0.041**
*B. Italy (STOXX Italy45 ESG)*				
*Constant*	0.010	0.000	38.25	0.000***
*|Rm,t|*	0.680	0.040	16.90	0.000***
*R*^*2*^*m,t*	1.626	0.739	2.201	0.027**
*D*^*cov*^* (R*^*2*^*m,.t)*	** − 2.068**	1.131	− 1.828	0.067*
*C. Greece (ATHEX ESG)*				
*Constant*	0.013	0.000	47.89	0.000***
*|Rm,t|*	0.503	0.022	22.53	0.000***
*R*^*2*^*m,t*	− 0.135	0.275	− 0.490	0.623
*D*^*cov*^* (R*^*2*^*m,.t)*	** − 1.943**	0.325	− 5.974	0.000***
*D. Spain (STOXX Spain30 ESG)*				
*Constant*	0.009	0.000	25.22	0.000***
*|Rm,t|*	0.514	0.072	7.094	0.000***
*R*^*2*^*m,t*	0.719	1.937	0.371	0.710
*D*^*cov*^* (R*^*2*^*m,.t)*	4.109	2.266	1.813	0.069*
*E. France (CAC40 ESG)*				
*Constant*	0.007	0.000	45.28	0.000***
*|Rm,t|*	0.156	0.036	4.302	0.000***
*R*^*2*^*m,t*	1.159	1.034	1.121	0.262
*D*^*cov*^* (R*^*2*^*m,.t)*	1.008	1.227	0.821	0.411
*F. Germany (DAX50 ESG)*				
*Constant*	0.007	0.000	54.02	0.000***
*|Rm,t|*	0.196	0.028	6.908	0.000***
*R*^*2*^*m,t*	0.111	0.896	0.124	0.900
*D*^*cov*^* (R*^*2*^*m,.t)*	0.845	0.768	1.100	0.271

Table 4 reports the estimated coefficients for the following model: $CSAD_{t} = \alpha + \gamma_{1} \left| {R_{m,t} } \right| + \gamma_{2} R_{m,t}^{2} + \gamma_{3} D^{{\text{cov}}} (R_{m,t}^{2} ) + \varepsilon_{t}$. *CSAD*_*t*_.is the cross-sectional absolute deviation of stock returns concerning cross sectional average return. *Rm,t. D*^*cov*^ is a dummy variable taking value 1 for covid-19 period (30 January 2020 to 01 June 2020) and 0 otherwise. *** Significant at 1%, ** Significant at 5% and * Significant at 10%. All estimations use the Newey and West (1987) heteroscedastic and autocorrelation corrected standards errors.

Our results indicate that Portugal, Italy and Greece are consistent with herding behavior during the Covid-19 period as there is a significantly negative coefficient *γ3*, Spain, France and Germany show no herding. Our results confirm Economou et al. (2011) who examined herd behavior in extreme market conditions for Greek, Italian, Portuguese, and Spanish stock markets for the period 1998–2008. Herding is found to be intense during periods of rising markets as the authors conclude (Economou et al. 2011). Our findings are consistent with Wu et al. (2020), who report significant herding in Chinese stocks during Covid-19. Moreover, our results partly confirm Rubbaniy et al. (2021) who disclose a significant herding behavior in the U.S. ESG leader stocks, during the Covid-19 outbreak. Furthermore, our finding is consistent with the Youssef et al. (2022) study which noticed the presence of herding during the COVID-19 crisis in the cryptocurrency market. On the other hand, our reported results are partly in contrast with Chang et al. (2000) who find no evidence of herding in a sample of energy markets from US, Europe, and Asia. Finally, we support Ciciretti et al. (2021) finding who detect an anti-herding behavior in ESG funds. Following our results, it seems that investors from Spain, France and Germany during the market crisis stay calm and select professional investment portfolio tools, in constructing investment portfolios by avoiding non-rational decisions, which occur due to herding behavior. The findings are robust under different time intervals with the same results, and therefore, offer useful information on herding behavior.

## Conclusions

Previous studies report a positive effect of ESG score and the financial performance of a firm, measured both by accounting and market-based indicators. A small number of studies show a negative relation, mainly for a single market. Furthermore, many studies report a non-significant relation. The current study aims to analyze a firm’s stock returns as a function of indicators, such as a firm’s size (Mcap), valuation (P/BV) ratio, risk (Sharpe) ratio and ESG score. To achieve this, we focus on six European countries. Our study contributes to relevant literature by providing evidence of the integration of market capitalization, price to book value, Sharpe ratio, stock returns and ESG performance of large-cap firms in Europe. Furthermore, it appears that higher inflows in certain asset classes are often the driving force behind herding behavior. Current investment strategies constantly focus on sustainability and, as a result, ESG investment is a strong candidate for the investigation of herd behavior. This motivates us to analyze whether ESG issues lead to herd behavior. The enlightenment of ESG herding improves not only investment decision-making, but also the process of constructing a well-diversified and optimized portfolio. However, despite the recent shift in the investment choice of global investors towards ESG stocks and the growing focus of academics on companies that adopt ESG practices, the existing literature has rarely highlighted the issue of herd investing in ESG stocks (Rubbaniy et al. 2021).

The results of this paper have several practical implications for the financial markets and their participants. First of all, the findings for five out of six European countries (Portugal, Greece, Spain, France and Germany) show that investors do not sacrifice their returns by investing in firms with high ESG scores, which means that sustainability concerns are not as high on the investor’s agenda. However, the benefit of not investing in highly ESG scoring firms might lead investors to select smaller size companies with a higher price to book value and higher Sharpe ratio as it is more likely to create higher returns. Our results offer new insights into investors and finance professionals by helping them to detect how financial (Mcap, P/BV, Sharpe ratio) and non-financial (ESG) factors might affect portfolio construction and performance. In particular, inexperienced investors and fund managers should be informed in more depth about ESG issues as it seems to play an important role within the sustainable investment frame.

Secondly, the findings are robust to Europe as a whole, using the Euronext100 large-cap index. Investors in Europe as whole sacrifice stock returns by buying companies that implement good ESG practices. This valuable and apropos result possibly means that European investors overall are socially responsible and implement portfolio diversification or long-run strategies. In addition, this novelty finding confirms the advancing strength of Europe as the biggest market for sustainable funds globally.

Thirdly, our results indicate that there is an interaction between ESG performance and herding in Greece and France. In addition, ESG investors in Portugal, Italy and Greece are involved in herd behavior during the Covid-19 crisis, which may result in market inefficiency and less diversified portfolios. ESG investors in Spain, France and Germany do not mimic the trading choices of others during the same period. This anti-herding behavior probably indicates that highly ESG scoring stocks conduce to market efficiency by lowering the probability of the financial bubble formation.

Lastly, the advantageous findings of this paper will help Asset Management Companies to understand the importance of herding behavior on ESG performance and take rational and profitable decisions. The results of this study offer useful policy implications in understanding the financial behavior drivers of market participants. Policymakers and regulators must ensure that all market participants should have free access to all relevant information, otherwise, investors and portfolio managers can emulate their compeer’s investment decisions leading to market anomalies.

Despite the valuable results of this study, there are also possible extensions for further work. In particular, the study panel should be expanded to more countries or continents. In addition, more financial stock indicators, such as price to earnings (P/E) ratio, price to cash flow (P/CF) ratio, volatility, return on equity (ROE) and return on asset (ROA), should be taken into consideration. Moreover, empirical analysis of the herding methodology utilizing the time-varying beta of herding through DCC-MIDAS (Dynamic conditional correlation with mixed data sampling) model could be used to strengthen behavior finance literature.

### Electronic supplementary material

Below is the link to the electronic supplementary material.

Supplementary file1 (ZIP 2622 kb)

## Appendix 1

### Supplementary data

Supplementary material related to this study can be found online at: https://drive.google.com/drive/folders/13V-rTp8d8jUvSxxgcGDpPQpviBP1iDfb
